# Supplementary material for: Community surveys of the prevalence, distribution, and coinfection of helminth and protozoan infections in semiurban and rural areas of Gabon, Central Africa
Source: PLoS Negl Trop Dis. 2025 Jun 12;19(6):e0013161. doi: 10.1371/journal.pntd.0013161 (PMC12161571; doi:10.1371/journal.pntd.0013161)
Supplement: S1 Table — (DOCX) [file pntd.0013161.s001.docx]

**S1 Table.** Distribution of protozoa species found during the survey other than *Cyclospora cayetanensis*, *Cystoisospora belli* and *Chilomastix* *mesnili* among the study population

|  | | | **N** | ***Entamoeba coli*** | | ***Giardia duodenalis*** | | ***Entamoeba H/D***** | | ***Endolimax Nana*** | | ***Iodomaeba butschili*** | | ***Blastocystis hominis*** | |
| --- | --- | --- | --- | --- | --- | --- | --- | --- | --- | --- | --- | --- | --- | --- | --- |
|  |  |  |  | **n, (%)** | **95%CI** | **n, (%)** | **95%CI** | **n, (%)** | **95%CI** | **n, (%)** | **95%CI** | **n, (%)** | **95%CI** | **n, (%)** | **95%CI** |
| **Overall** | | | 944 | 80, (8.5) | 6.8 – 10.4 | 27, (2.9) | 1.9 – 4.1 | 27, (2.9) | 1.9 – 4.1 | 31, (3.3) | 2.2 – 4.6 | 38, (4.0) | 2.9 – 5.5 | 101, (10.7) | 8.8 – 12.8 |
| **Age range** | | |  |  |  |  |  |  |  |  |  |  |  |  |  |
|  | 1 – 4 | | 88 | 2, (2.3) | 0.3 – 8.0 | 5, (5.7) | 1.9 – 12.8 | 0, - | - | 1, (1.1) | 0.0 – 6.2 | 2, (2.3) | 0.3 – 8.0 | 6, (6.8) | 2.5 – 14.2 |
|  | 5 – 19 | | 306 | 28, (9.1) | 6.2 – 12.9 | 11, (3.6) | 1.8 – 6.3 | 6, (2.0) | 0.7 – 4.2 | 12, (3.9) | 2.0 – 6.7 | 13, (4.2) | 2.3 – 7.1 | 39, (12.7) | 9.2 – 17.0 |
|  | 20 – 49 | | 266 | 16, (6.0) | 3.5 – 9.6 | 5, (1.9) | 0.6 – 4.3 | 7, (2.6) | 1.1 – 5.3 | 11, (4.1) | 2.1 – 7.3 | 12, (4.5) | 2.3 – 7.7 | 25, (9.4) | 6.2 – 13.6 |
|  | ≥ 50 | | 284 | 34, (12.0) | 8.4 – 16.3 | 6, (2.1) | 0.8 – 4.5 | 14, (4.9) | 2.7 – 8.1 | 7, (2.5) | 1.0 – 5.0 | 11, (3.9) | 1.9 – 6.8 | 31, (10.9) | 7.5 – 15.1 |
| **Gender** | | |  |  |  |  |  |  |  |  |  |  |  |  |  |
|  | Female | | 515 | 40, (7.8) | 5.6 – 10.4 | 14, (2.7) | 1.5 – 4.5 | 11, (2.1) | 1.1 – 3.8 | 16, (3.1) | 1.8 – 5.0 | 19, (3.7) | 2.2 – 5.7 | 49, (9.5) | 7.1 – 12.4 |
|  |  | WRA | 183 | 8, (4.4) | 1.9 – 8.4 | 3, (1.6) | 0.3 – 4.7 | 3, (1.6) | 0.3 – 4.7 | 6, (3.3) | 1.2 – 7.0 | 8, (4.4) | 1.9 – 8.4 | 19, (10.4) | 6.4 – 15.7 |
|  | Male | | 429 | 40, (9.3) | 6.7 – 12.5 | 13, (3.0) | 1.6 – 5.1 | 16, (3.7) | 2.1 – 6.0 | 15, (3.5) | 2.0 – 5.7 | 19, (4.4) | 2.7 – 6.8 | 52, (12.1) | 9.2 – 15.6 |
| **Location** | | |  |  |  |  |  |  |  |  |  |  |  |  |  |
|  | Lambaréné | | 405 | 17, (4.2) | 2.5 – 6.6 | 13, (3.2) | 1.7 – 5.4 | 13, (3.2) | 1.7 – 5.4 | 21, (5.2) | 3.2 – 7.8 | 14, (3.5) | 1.9 – 5.7 | 61, (15.1) | 11.7 – 18.9 |
|  | Southern rural area | | 194 | 29, (14.9) | 10.2 – 20.8 | 7, (3.6) | 1.5 – 7.3 | 5, (2.6) | 0.8 – 5.9 | 5, (2.6) | 0.8 – 5.9 | 18, (9.3) | 5.6 – 14.3 | 19, (9.8) | 6.0 – 14.9 |
|  | Northern rural area | | 345 | 34, (9.8) | 6.9 – 13.5 | 7, (2.0) | 0.8 – 4.1 | 9, (2.6) | 1.2 – 4.9 | 5, (1.4) | 0.5 – 3.3 | 6, (1.7) | 0.6 – 3.7 | 21, (6.1) | 3.8 – 9.1 |
| **Occupation** | | |  |  |  |  |  |  |  |  |  |  |  |  |  |
|  | No occupation | | 240 | 15, (6.2) | 3.5 – 10.1 | 7, (2.9) | 1.2 – 5.9 | 5, (2.1) | 0.7 – 4.8 | 9, (3.7) | 1.7 – 7.0 | 7, (2.9) | 1.2 – 5.9 | 24, (10.0) | 6.5 – 14.5 |
|  | Farmer | | 230 | 32, (13.9) | 9.7 – 19.1 | 5, (2.2) | 0.7 – 5.0 | 11, (4.8) | 2.4 – 8.4 | 3, (1.3) | 0.3 – 3.8 | 11, (4.8) | 2.4 – 8.4 | 17, (7.4) | 4.4 – 11.6 |
|  | Students | | 337 | 28, (8.3) | 5.6 – 11.8 | 11, (3.3) | 1.6 – 5.8 | 7, (2.1) | 0.8 – 4.2 | 13, (3.9) | 2.1 – 6.5 | 13, (3.9) | 2.1 – 6.5 | 43, (12.8) | 9.4 – 16.8 |
|  | Others | | 137 | 5, (3.6) | 1.2 – 8.3 | 4, (2.9) | 0.8 – 7.3 | 4, (2.9) | 0.8 – 7.3 | 6, (4.4) | 1.6 – 9.3 | 7, (5,1) | 2.1 – 10.2 | 17, (12.4) | 7.4 – 19.1 |

**Histolitica/Dispar
