# Supplementary material for: Community surveys of the prevalence, distribution, and coinfection of helminth and protozoan infections in semiurban and rural areas of Gabon, Central Africa
Source: PLoS Negl Trop Dis. 2025 Jun 12;19(6):e0013161. doi: 10.1371/journal.pntd.0013161 (PMC12161571; doi:10.1371/journal.pntd.0013161)
Supplement: S2 Table — (DOCX) [file pntd.0013161.s002.docx]

**S2 Table.** List of combinations of helminth and protozoan coinfections

| **Type of co-infection** | | | **n** | **%** |
| --- | --- | --- | --- | --- |
| **Mono infection** | | | **341** | **58.3** |
|  |  | *Ascaris lumbricoides* | 9 | 2.6 |
|  |  | *Blastocystis hominis* | 48 | 14.1 |
|  |  | *Cyclospora cayetanensis* | 5 | 1.5 |
|  |  | *Entamoeba histolytica/dispar* | 6 | 1.8 |
|  |  | *Entamoeba coli* | 29 | 8.5 |
|  |  | *Endolimax nana* | 14 | 4.1 |
|  |  | *Giardia duodenalis* | 11 | 3.2 |
|  |  | Hookworm | 17 | 5.0 |
|  |  | *Iodamoeba butschlii* | 6 | 1.8 |
|  |  | *Loa-loa* | 28 | 8.2 |
|  |  | *Mansonella perstans* | 5 | 1.5 |
|  |  | *Plasmodium sp.* | 63 | 18.5 |
|  |  | *Schistosoma haematobium* | 56 | 16.4 |
|  |  | *Strongyloides stercoralis* | 10 | 2.9 |
|  |  | *Trichuris trichiura* | 34 | 10.0 |
| **Co-infection** | | | **244** | **41.7** |
|  | **Infection with two parasitic species** | | **152** | **62.3** |
|  |  | *A. lumbricoides, B. hominis* | 1 | 0.7 |
|  |  | *A. lumbricoides, E. coli* | 1 | 0.7 |
|  |  | *A. lumbricoides, I. butschlii* | 1 | 0.7 |
|  |  | *A. lumbricoides, T. trichiura* | 5 | 3.3 |
|  |  | *B. hominis, Chilomastix mesnili** | 2 | 1.3 |
|  |  | *B. hominis, E. coli* | 1 | 0.7 |
|  |  | *B. hominis, E. histolytica/dispar* | 1 | 0.7 |
|  |  | *B. hominis, En. nana* | 2 | 1.3 |
|  |  | *B. hominis, G. duodenalis* | 1 | 0.7 |
|  |  | *B. hominis, I. butschlii* | 2 | 1.3 |
|  |  | *E. coli, E. histolytica/dispar* | 3 | 2.0 |
|  |  | *E. coli, G. duodenalis* | 2 | 1.3 |
|  |  | *E. coli, I. butschlii* | 1 | 0.7 |
|  |  | *E. histolytica/dispar, I. butschlii* | 2 | 1.3 |
|  |  | *En. nana, E. coli* | 3 | 2.0 |
|  |  | *En. nana, E. histolytica/dispar* | 1 | 0.7 |
|  |  | Hookworm*, B. hominis* | 3 | 2.0 |
|  |  | Hookworm*, E. coli* | 4 | 2.6 |
|  |  | Hookworm*, E. histolytica/dispar* | 1 | 0.7 |
|  |  | Hookworm*, I. butschlii* | 1 | 0.7 |
|  |  | *Hookworm, St. stercoralis* | 2 | 1.3 |
|  |  | *Loa-loa, A. lumbricoides* | 1 | 0.7 |
|  |  | *Loa-loa, B. hominis* | 3 | 2.0 |
|  |  | *Loa-loa, E. coli* | 3 | 2.0 |
|  |  | *Loa-loa, E. histolytica/dispar* | 1 | 0.7 |
|  |  | *Loa-loa,* Hookworm | 5 | 3.3 |
|  |  | *Loa-loa, M. perstans* | 4 | 2.6 |
|  |  | *Loa-loa, S. haematobium* | 2 | 1.3 |
|  |  | *Loa-loa, T. trichiura* | 3 | 2.0 |
|  |  | *M. perstans, A. lumbricoides* | 1 | 0.7 |
|  |  | *M. perstans, B. hominis* | 1 | 0.7 |
|  |  | *M. perstans,* Hookworm | 1 | 0.7 |
|  |  | *M. perstans, T. trichiura* | 1 | 0.7 |
|  |  | *Plasmodium sp., A. lumbricoides* | 2 | 1.3 |
|  |  | *Plasmodium sp., B. hominis* | 7 | 4.6 |
|  |  | *Plasmodium sp., Cystoisospora belli** | 1 | 0.7 |
|  |  | *Plasmodium sp., E. coli* | 5 | 3.3 |
|  |  | *Plasmodium sp., E. histolytica/dispa* | 2 | 1.3 |
|  |  | *Plasmodium sp., En. nana* | 2 | 1.3 |
|  |  | *Plasmodium sp., G. duodenalis* | 1 | 0.7 |
|  |  | *Plasmodium sp.,* Hookworm | 3 | 2.0 |
|  |  | *Plasmodium sp., I. butschlii* | 4 | 2.6 |
|  |  | *Plasmodium sp., Loa-loa* | 2 | 1.3 |
|  |  | *Plasmodium sp., M. perstans* | 3 | 2.0 |
|  |  | *Plasmodium sp., S. haematobium* | 9 | 5.9 |
|  |  | *Plasmodium sp., T. trichiura* | 6 | 3.9 |
|  |  | *S. haematobium, A. lumbricoides* | 1 | 0.7 |
|  |  | *S. haematobium, B. hominis* | 7 | 4.6 |
|  |  | *S. haematobium, Cyclospora cayetanensiss* | 1 | 0.7 |
|  |  | *S. haematobium, E. coli* | 2 | 1.3 |
|  |  | *S. haematobium, En. nana* | 2 | 1.3 |
|  |  | *S. haematobium, G. duodenalis* | 2 | 1.3 |
|  |  | *S. haematobium,* Hookworm | 3 | 2.0 |
|  |  | *S. haematobium, I. butschlii* | 3 | 2.0 |
|  |  | *St. stercoralis, E. coli* | 1 | 0.7 |
|  |  | *T. trichiura, B. hominis* | 1 | 0.7 |
|  |  | *T. trichiura, Cyclospora cayetanensis* | 1 | 0.7 |
|  |  | *T. trichiura, E. coli* | 2 | 1.3 |
|  |  | *T. trichiura, G. duodenalis* | 1 | 0.7 |
|  |  | *T. trichiura,* Hookworm | 11 | 7.2 |
|  |  | *T. trichiura, St. stercoralis* | 1 | 0.7 |
|  | **Infection with three parasitic species** | | **68** | **27.9** |
|  |  | *A. lumbricoides, B. hominis, I. butschlii* | 1 | 1.5 |
|  |  | *A. lumbricoides, E. coli, I. butschlii* | 1 | 1.5 |
|  |  | *A. lumbricoides, T. trichiura, B. hominis* | 1 | 1.5 |
|  |  | *A. lumbricoides, T. trichiura,* Hookworm | 5 | 7.4 |
|  |  | *A. lumbricoides, T. trichiura, St. stercoralis* | 2 | 2.9 |
|  |  | *E. coli, G. duodenalisa, I. butschlii* | 1 | 1.5 |
|  |  | *En. nana, E. coli, I. butschlii* | 1 | 1.5 |
|  |  | Hookworm*, B. hominis, Chilomastix mesnili** | 1 | 1.5 |
|  |  | Hookworm*, B. hominis, E. histolytica/dispar* | 1 | 1.5 |
|  |  | Hookworm*, E. histolytica/dispar, I. butschlii* | 1 | 1.5 |
|  |  | *Loa-loa, Hookworm, St. stercoralis* | 2 | 2.9 |
|  |  | *Loa-loa, M. perstans,* Hookworm | 3 | 4.4 |
|  |  | *Loa-loa, M. perstans, T. trichiura* | 1 | 1.5 |
|  |  | *Loa-loa, S. haematobium, B. hominis* | 1 | 1.5 |
|  |  | *Loa-loa, T. trichiura, B. hominis* | 1 | 1.5 |
|  |  | *Loa-loa, T. trichiura,* Hookworm | 2 | 2.9 |
|  |  | *M. perstans, S. haematobium, T. trichiura* | 1 | 1.5 |
|  |  | *M. perstans, T. trichiura,* Hookworm | 1 | 1.5 |
|  |  | *Plasmodium sp., A. lumbricoides, B. hominis* | 1 | 1.5 |
|  |  | *Plasmodium sp., A. lumbricoides, G. duodenalis* | 1 | 1.5 |
|  |  | *Plasmodium sp., A. lumbricoides, I. butschlii* | 1 | 1.5 |
|  |  | *Plasmodium sp., A. lumbricoides, T. trichiura* | 1 | 1.5 |
|  |  | *Plasmodium sp., B. hominis, I. butschlii* | 1 | 1.5 |
|  |  | *Plasmodium sp., E. coli, G. duodenalis* | 1 | 1.5 |
|  |  | *Plasmodium sp., Loa-loa, E. coli* | 1 | 1.5 |
|  |  | *Plasmodium sp., Loa-loa,* Hookworm | 1 | 1.5 |
|  |  | *Plasmodium sp., Loa-loa, S. haematobium* | 1 | 1.5 |
|  |  | *Plasmodium sp., Loa-loa, T. trichiura* | 2 | 2.9 |
|  |  | *Plasmodium sp., M. perstans, A. lumbricoides* | 1 | 1.5 |
|  |  | *Plasmodium sp., M. perstans, E. coli* | 1 | 1.5 |
|  |  | *Plasmodium sp., M. perstans, T. trichiura* | 1 | 1.5 |
|  |  | *Plasmodium sp., S. haematobium, B. hominis* | 1 | 1.5 |
|  |  | *Plasmodium sp., S. haematobium, E. coli* | 1 | 1.5 |
|  |  | *Plasmodium sp., S. haematobium, G. duodenalis* | 1 | 1.5 |
|  |  | *Plasmodium sp., S. haematobium, T. trichiura* | 1 | 1.5 |
|  |  | *Plasmodium sp., T. trichiura, E. coli* | 1 | 1.5 |
|  |  | *Plasmodium sp., T. trichiura,* Hookworm | 1 | 1.5 |
|  |  | *S. haematobium, B. hominis, E. histolytica/dispar* | 1 | 1.5 |
|  |  | *S. haematobium, B. hominis, En. nana* | 1 | 1.5 |
|  |  | *S. haematobium, B. hominis, I. butschlii* | 1 | 1.5 |
|  |  | *S. haematobium, E. coli, I. butschlii* | 2 | 2.9 |
|  |  | *S. haematobium, E. histolytica/dispar, I. butschlii* | 1 | 1.5 |
|  |  | *S. haematobium, En. nana, E. coli* | 1 | 1.5 |
|  |  | *S. haematobium,* Hookworm*, En. nana* | 1 | 1.5 |
|  |  | *S. haematobium,* Hookworm*, G. duodenalis* | 1 | 1.5 |
|  |  | *S. haematobium, Hookworm, St. stercoralis* | 1 | 1.5 |
|  |  | *S. haematobium, T. trichiura, B. hominis* | 1 | 1.5 |
|  |  | *S. haematobium, T. trichiura, E. coli* | 1 | 1.5 |
|  |  | *St. stercoralis, En. nana, E. coli* | 1 | 1.5 |
|  |  | *T. trichiura, B. hominis, I. butschlii* | 1 | 1.5 |
|  |  | *T. trichiura,* Hookworm*, B. hominis* | 1 | 1.5 |
|  |  | *T. trichiura,* Hookworm*, Cystoisospora belli** | 1 | 1.5 |
|  |  | *T. trichiura,* Hookworm*, E. coli* | 1 | 1.5 |
|  |  | *T. trichiura,* Hookworm*, E. histolytica/dispar* | 1 | 1.5 |
|  |  | *T. trichiura,* Hookworm*, En. nana* | 1 | 1.5 |
|  |  | *T. trichiura, Hookworm, St. stercoralis* | 2 | 2.9 |
|  | **Infection with four parasitic species** | | **16** | **6.6** |
|  |  | *A. lumbricoides, T. trichiura,* Hookworm*, G. duodenalis* | 1 | 11.1 |
|  |  | *Loa-loa, M. perstans, Hookworm, St. stercoralis* | 1 | 11.1 |
|  |  | *Loa-loa, M. perstans, T. trichiura,* Hookworm | 1 | 11.1 |
|  |  | *M. perstans, T. trichiura,* Hookworm*, G. duodenalis* | 1 | 11.1 |
|  |  | *Plasmodium sp., B. hominis, s histo, I. butschlii* | 1 | 11.1 |
|  |  | *Plasmodium sp.,* Hookworm*, E. coli, E. histolytica/dispar* | 1 | 11.1 |
|  |  | *Plasmodium sp., Loa-loa, A. lumbricoides, E. coli* | 1 | 11.1 |
|  |  | *Plasmodium sp., Loa-loa, E. histolytica/dispar, Giargia duodenalis* | 1 | 11.1 |
|  |  | *Plasmodium sp., S. haematobium, A. lumbricoides, T. trichiura* | 1 | 11.1 |
|  |  | *Plasmodium sp., S. haematobium, B. hominis, Cystoisospora belli** | 1 | 11.1 |
|  |  | *Plasmodium sp., S. haematobium, B. hominis, E. coli* | 1 | 11.1 |
|  |  | *Plasmodium sp., T. trichiura,* Hookworm*, E. coli* | 1 | 11.1 |
|  |  | *Plasmodium sp., T. trichiura,* Hookworm*, I. butschlii* | 1 | 11.1 |
|  |  | *S. haematobium, A. lumbricoides, T. trichiura, E. coli* | 1 | 11.1 |
|  |  | *S. haematobium, T. trichiura, B. hominis, En. nana* | 1 | 11.1 |
|  |  | *T. trichiura, Hookworm, St. stercoralis, E. coli* | 1 | 11.1 |
|  | **Infection with five parasitic species** | | **6** | **2.5** |
|  |  | *Loa-loa, M. perstans, A. lumbricoides, B. hominis, I. butschlii* | 1 | 16.7 |
|  |  | *Loa-loa, T. trichiura, Hookworm, St. stercoralis, I. butschlii* | 1 | 16.7 |
|  |  | *Plasmodium sp., A. lumbricoides, T. trichiura,* Hookworm*, B. hominis* | 1 | 16.7 |
|  |  | *Plasmodium sp., T. trichiura, Hookworm, St. stercoralis, E. coli* | 1 | 16.7 |
|  |  | *S. haematobium, A. lumbricoides, T. trichiura, E. coli, E. histolytica/dispar* | 1 | 16.7 |
|  |  | *T. trichiura, Hookworm, St. stercoralis, E. histolytica/dispar, I. butschlii* | 1 | 16.7 |
|  | **Infection with six parasitic species** | | **2** | **0.8** |
|  |  | *Loa-loa, M. perstans, T. trichiura, Hookworm, St. stercoralis, I. butschlii* | 1 | 50.0 |
|  |  | *Plasmodium sp., Loa-loa, A. lumbricoides, T. trichiura,* Hookworm | 1 | 50.0 |

*Present only in coinfection
