# Supplementary material for: Community surveys of the prevalence, distribution, and coinfection of helminth and protozoan infections in semiurban and rural areas of Gabon, Central Africa
Source: PLoS Negl Trop Dis. 2025 Jun 12;19(6):e0013161. doi: 10.1371/journal.pntd.0013161 (PMC12161571; doi:10.1371/journal.pntd.0013161)
Supplement: Attachment — Submitted filename: Response to Reviewers.docx [file pntd.0013161.s005.docx]

**Community surveys of the prevalence, distribution, and coinfection of helminth and protozoan infections in semiurban and rural areas of Gabon, Central Africa**

Date: May 7^th^, 2025

Dear Editors of PLOS Neglected Tropical Diseases,

We are grateful for the consideration of our manuscript to your journal, as well as for the scientific editor’s and reviewer’s comments and recommendations. We have replied to all the recommendations and have modified the manuscript accordingly. We are convinced that these recommendations improved the quality of our manuscript.

Please find below our responses to the scientific editor’s and reviewer’s comments.

With kind regards,

Jean Claude Dejon Agobé

Corresponding author

**Scientific editor:**

The manuscript “Community surveys of the prevalence, distribution, and coinfection of helminth and protozoan infections in semiurban and rural areas of Gabon, Central Africa” by Dejon Agobé et al. is a cross-sectional study of 1,084 participants, aimed to describe the distribution of helminth and protozoan infections in the Moyen-Ogooué province of Gabon, where there is near 70,000 inhabitants. The results indicated a moderate prevalence of helminths and protozoa in that community, with age, gender, and location playing a significant role in the parasitic distribution. Although some parasite intensity of infection was mostly light, the intensity of *Schistosoma haematobium* was heavy in 35% of the cases, which is of concern, particularly when affected women of reproductive age that can have female genital schistosomiasis. A high number of different helminths and protozoa (3 participants were infected with six different species), parasites were observed in coinfections. It is the first study in that province in Gabon. Previous studies were performed in other provinces of Gabon (references 24 and 31, line 429, also reference 17). It is of interest that lower parasites seem to be related to the widespread use of anthelmintics in the country. The cross-sectional study was conducted with great care, including sample size calculation and sampling procedures.

Lines 132-133. Did the authors analyze the presence of parasites in relation to the rainy seasons (2) or dry seasons (2)?

**Reply: We thank the editor for this question. Although the participants were recruited over a year, we did not consider the seasons in our analysis. We agree that such an analysis could be relevant for some parasites, such as *Plasmodium* spp., and for other infections, which are very often chronic. The impact of seasons on their prevalence will be difficult to assess in a cross-sectional study design, as the time of infection could be difficult to determine. However, it should be noted that seasonality effects are limited in view of perennially high temperatures and humidity; this being the reason why we refrained from such an analysis in the first place.**

Line 137. Clarify that the semi urban area mentioned for Lambaréné is area 1 of the study.

**Reply: We thank the reviewer for this query. There are three study areas, the semi-urban area of Lambaréné, and then the northern and southern rural areas along the Route Nationale 1 (N1) to the north and south, as explained from lines 137 onwards and as summarised in Table 1.**

Line 147. The considered prevalence of 50% was higher than that observed. Would that modify the number of samples needed? Since the study collected much more than the samples needed, this is just a comment for personal interest.

**Reply: We thank the editor for this comment. We choose to consider 50% prevalence for the calculation of the sample size, as it yields the maximum calculated sample size via Daniel’s formula. Using more or less than 50% prevalence will yield a required sample size less than the 272 we found.**

Section on laboratory analysis, could give some more details on the specific methods used. As indicated all the analysis were based only on microscopy.

**Reply: We thank the editor for the suggestion. We have added details about the laboratory analysis used in the study.**

Any staining techniques performed to visualized acid-alcohol resistant protozoa?

**Reply: We thank the editor for this question. We did not use staining techniques to visualize acid- alcohol-resistant protozoa.**

Line 183- The text mentions “coproculture” for hookworms. Were any hookworms identified to the genera/species level?

**Reply: We thank the editor for this question. Hookworm larvae were indeed identified via coproculture and the Harada‒Mori technique. This approach has improved the sensitivity of the method we used for the diagnosis of hookworm infection. In addition to the 82 participants who were positive for the presence of hookworm eggs in their stool via the Kato-Katz technique, 10 more participants were positive for only the presence of hookworm larvae. This last information has been added to the revised version of the manuscript.**

Line 182. Spell out TBS.

**Reply: We thank the editor for the suggestion. TBS is spelled out in line 172, where it was used for the first time.**

Statistical consideration. The tests used for statistical analysis could be indicated. E.g. anova, t-test, Chi-square….

**Reply: We thank the editor for the suggestion. Our analysis was basically descriptive. We therefore presented only a summary of our data. However, the difference in the level of proportion was determined under the assumption that no overlapping confidence intervals indicate statistical significance. We added this information to the revised version of the manuscript from lines 204-207.**

Line 236. Were the two cases of Enterobius vermicularis related to age?

**Reply: We thank the editor for this question. Enterobiasis vermicularis was not related to age. One of the two participants was 9 years old, and the other was 55 years old. We added this information to the revised version of the manuscript.**

Tables. Authors could add an asterisk in those parasites and factors that were statistically significant to easier understanding of differences.

**Reply: We thank the editor for the suggestion. As indicated earlier in the letter, we used confidence intervals to assess the difference between proportions. As we did not set a reference modality when comparing proportions, particularly for variables with more than two modalities, the use of asterisks to indicate statistically significant differences between proportions became difficult.**

Titles of lines 245, 260 and 272. Please change “Distribution” by “Prevalence”.

**Reply: We thank the editor for the suggestion. We changed “Distribution” to “Prevalence” in the title of Tables 2 and 3.**

Lines 256-257 and lines 257-258. Please include *p-values* in the results statistically significant. Same for other sections.

**Reply: We thank the editor for the suggestion. We did not perform a statistical test to compare proportions. As we previously indicated, we used the assumption that no overlapping confidence intervals indicate statistical significance in differences in proportion levels.**

Lines 278 and 280. “More prevalent” but statistically significant?

**Reply: We thank the editor for this remark. The difference in the schistosomiasis prevalence level between study areas is indeed statistically significant. The correction was made accordingly.**

Line 283. Modify “trichuriasis” by “trichiura”

**Reply: We thank the editor for this remark. We corrected “trichuriasis” to “trichiura”.**

Line 285. The heaviest infections by *S. haematobium* seemed to occur in age 1-4 years and older than 50 years. Is this correct? Please indicate in text.

**Reply: We thank the editor for the suggestion. We have indicated in the revised version of the manuscript that participants aged 1--4 years and those aged 50 years and over represent a greater proportion of heavy infections.**

Line 309. Higher prevalence, but statistically significant?

**Reply: We thank the editor for this remark. We have indicated in the text the statistically significant difference between the two prevalence values, which is determined here on the basis of the absence of overlap in both confidence intervals.**

Line 322. Where filarial infections found in areas where their vectors are more frequently found?

**Reply: We thank the editor for this question. The link between the distribution of filarial infection in our study population and the vector in the region is difficult to establish, as no such study has yet been conducted in our region. Empirically, the two rural areas are known to be more endemic for the filariasis vector than Lambaréné is, although within Lambaréné itself, some parts of the town are suspected of being at higher risk than others are.**

The discussion is sometimes a repetition of the results and could be reduced.

**Reply: We thank the editor for the suggestion. To compare our findings with those of some previous studies, we sometimes needed to recall our results, with the idea that this could help with reading. The objective is to facilitate reading.**

Line 396. Change “aera” by “area”.

**Reply: We thank the editor for this remark. The correction has been made accordingly.**

Line 449. Indicate the common risk factors for those parasites.

**Reply: We thank the editor for the suggestion. Although investigations are needed to identify risk factors, we believe that environmental factors are basically the main common factors shared by *Plasmodium* sp., *S. haematobium*, and *T. trichiura*, which can explain the high occurrence of coinfection. We added this latest information to the revised version of the manuscript.**

The title of Supplementary table S1 do not correspond to the data shown in that table.

**Reply: We thank the editor for this remark. The title of S1 Table has been corrected.**

**Reviewer #1**

The study has provided epidemiological data on helminth and protozoan infection in the community. Authors need to do minor corrections.

Line 86- Remove ‘for example’ in the sentence

**Reply: We thank the reviewer for this suggestion. We removed “for example” from the sentence.**

Line 93- spelling error – platyhelminths instead of plathelminths

**Reply: We thank the reviewer for this remark. The correction was made accordingly.**

Line 102- spelling error – mastigophora instead of mastigora

**Reply: We thank the reviewer for this remark. We corrected this word accordingly.**

Line 119- correct the cause of filariasis. It is caused by Wuchereria bancrofti, Brugia malayi and Brugia timori, not Onchocerca volvolus.

**Reply: We thank the reviewer for this suggestion. We chose to indicate onchocerciasis as filariasis, as it is endemic in Gabon (the study area), in contrast to lymphatic filariasis, which is not endemic in Central Africa (which includes Gabon).**

Line 144- State the exclusive criteria in the study

**Reply: We thank the reviewer for this suggestion. We did not have exclusion criteria. All volunteers for whom we obtained signed informed consent were assessed according to the inclusion criteria.**

In Table 1- Female to sex ratio should be 1:15 not 1.15

**Reply: We thank the reviewer for this suggestion. However, as we provided a ratio of 579:505, we believe that 1.15 is appropriate for expressing the sex ratio.**

Line 228-229 – The prevalence of 36% (357/980, 95% CI: 18 – 23) NOT ‘IC’ should be stated in Fig 2 under the total number for helminths as this is not seen anywhere in the tables. This should also be done for Protozoans.

**Reply: We thank the reviewer for the remark and suggestion. The term “CI” was corrected. However, it will be difficult to indicate the 36% prevalence of any helminth infection in Figure 2 as this include only participant positive either for helminth or protozoa infections.**

Line 231 – Insert Table 3- after (94/1084,95%CI: 7-10) Table 3.

**Reply: We thank the reviewer for this suggestion. We revised the paragraph and indicated Table 3 as a reference for the results presented in the text.**

Line 247 – Prevalence rate was rounded up to 8% instead of 8.3%. I suggest Prevalence rates be left as it is.

**Reply: We thank the reviewer for this suggestion. We indeed rounded all the numbers in the text. We want to keep the results as they are because we think it makes the results easier to read.**

Line 265 – Prevalence rate is 9.4% not 8% as written

**Reply: We thank the reviewer for this remark. The prevalence rate of Loa-loa for males has been corrected.**

Line 266 – Prevalence rate of M. perstans is 4.2% for males vs 1.6% for females

**Reply: We thank the reviewer for this comment. We hope to understand this request. In parentheses for *Loa loa* and *M. perstans*, we have indicated in the revised version of the manuscript first the prevalence in females, which served here as the reference modality, and then the prevalence in males.**

Line 278 – Prevalence of 18% not 16%

**Reply: We thank the reviewer for this remark. The correction has been made in the manuscript accordingly.**

Line 279 – Prevalence of 5% and not 16%

**Reply: We thank the reviewer for this remark. The corrections have been made in the revised version of the manuscript for the prevalence of schistosomiasis in Lambaréné and in the northern rural area.**

Line 291 – 293 There is no information in Table 5 that gives the data of 28 (20%) infected participants presenting with fever at the time of inclusion and 34 (24%) reporting a history of fever over the 3 past days from the day of inclusion. Is there a supporting document for this?

**Reply: We thank the reviewer for this question. We revised the sentence and tried to make it clear that the information is not available in Table 5. Indeed, there is no supporting document or Table for these data. We provided that data in the text as additional information, as one can be interested in the number of participants with fever among those positive for *Plasmodium* parasites. Malaria indeed remains of particular interest in our community.**

Line 294 – 300 talks about the different parasites as shown in Figure 2. Insert Fig 2 after the sentence in in line 294 for clarification.

**Reply: We thank the reviewer for this suggestion. We have reorganized the paragraph indicating in which table or figure the data we present are located.**

Line 305 – 310 contains information in Table 5. Insert Table 5 to indicate this.

**Reply: We thank the reviewer for this remark. Table 5 is indicated at the beginning of the section.**

Line 330 – 341 has no Table or Figure to support the information given about Helminths and Protozoan coinfections. A table should be included for this.

**Reply: We thank the reviewer for this suggestion. We have added a table on coinfection. Please see Supplementary Table S2. We took advantage of this comment to reconsider part of the results of this section and present the number of oc6c6urrences of the most common parasites in coinfection instead of the number of coinfection combinations in which the “most common parasites” were involved.**

Fig.6 is not clear. There is a need for a sharp and clearer figure

**Reply: We thank the reviewer for this remark. Fig. 6 has been revised.**

Line 499 – Insert the names of the authors in the reference

**Reply: We thank the reviewer for this suggestion. The reference has been corrected accordingly.**
